# Assessing the long-term effects of zero-tillage on the macroporosity of Brazilian soils using X-ray Computed Tomography

**DOI:** 10.1016/j.geoderma.2018.11.031

**Published:** 2019-03-01

**Authors:** M.V. Galdos, L.F. Pires, H.V. Cooper, J.C. Calonego, C.A. Rosolem, S.J. Mooney

**Affiliations:** a Institute for Climate and Atmospheric Science, School of Earth and Environment, University of Leeds, Leeds LS2 9JT, UK; b Department of Physics, State University of Ponta Grossa, Ponta Grossa, Brazil; c Division of Agricultural & Environmental Science, University of Nottingham, Sutton Bonington Campus, UK; d Department of Crop Science, São Paulo State University, Botucatu, Brazil

**Keywords:** Zero tillage, Macroporosity, Pore connectivity, Network tortuosity, Pore shape

## Abstract

Zero-tillage (ZT) is being increasingly adopted globally as a conservationist management system due to the environmental and agronomic benefits it provides. However, there remains little information on the tillage effect on soil pore characteristics such as shape, size and distribution, which in turn affect soil physical, chemical and biological processes. X-ray micro Computed Tomography (μCT) facilitates a non-destructive method to assess soil structural properties in three-dimensions. We used X-ray μCT at a resolution of 70 μm to assess and calculate the shape, size and connectivity of the pore network in undisturbed soil samples collected from a long-term experiment (~30 years) under zero tillage (ZT) and conventional tillage (CT) systems in Botucatu, Southeastern Brazil. In both systems, a single, large pore (>1000 mm^3^) typically contributed to a large proportion of macroporosity, 91% in CT and 97% in ZT. Macroporosity was higher in ZT (19.7%) compared to CT (14.3%). However the average number of pores was almost twice in CT than ZT. The largest contribution in both treatments was from very complex shaped pores, followed by triaxial and acircular shaped. Pore connectivity analysis indicated that the soil under ZT was more connected that the soil under CT. Soil under CT had larger values of tortuosity than ZT in line with the connectivity results. The results from this study indicate that long-term adoption of ZT leads to higher macroporosity and connectivity of pores which is likely to have positive implications for nutrient cycling, root growth, soil gas fluxes and water dynamics.

## Highlights

• Soil macro-porosity was higher under zero-tillage than conventional tillage.
• Conventional tillage increases the number of pores but decreases the connectivity between them.
• Zero-tillage presented lower values of pore tortuosity, indicating better connectivity.
• Long term zero tillage improves soil pore connectivity.
• 3D analyses of pore shape indicated difference between tillage systems.

## 1 Introduction

Zero-tillage (ZT), where the seed is sown directly into the soil causing minimal disturbance and retaining surface crop residue, has been widely adopted since the 1940s with 111 million ha managed globally using this approach (Derpsch et al., 2010). Studies on the effects of ZT on soil properties have previously shown increases in water infiltration rate and storage capacity (Copec et al., 2015; Su et al., 2007), and decreases in surface runoff and erosion compared with conventionally tilled (CT) soils (DeLaune et al., 2013). These differences are ascribed to an increase in aggregate stability, higher numbers of biopores originating from earthworms and root growth and a decrease in the frequency of machinery traffic passing over the soil, which collectively alters soil porosity (Haghighi et al., 2010). Soil porosity is an important physical attribute of a soil's structure as it determines the oxygen and water availabilities, which in turn affects gas exchange and crop yield (Franzluebbers, 2002).

Additionally, there remains a lack of knowledge regarding the effect of undisturbed soil structure on functional processes. A greater understanding of the role of structure on soil function can be achieved by a detailed characterisation of the spatial configuration of its components. In the context of global environmental change and agricultural expansion, there is an urgent need to assess how ZT, and in particular how this changes over the time-scale of years or decades, impacts on soil pore shape, size and connectivity. ZT systems have been associated with significant changes in soil porosity, especially in the upper few centimetres in comparison to CT soils (Anikwe and Ubochi, 2007). Sasal et al. (2006) concluded that soil porosity in CT, which was calculated from bulk density and measured particle density, was 3.5% higher than under ZT in surface soils (0–15 cm). Mangalassery et al. (2014) found larger differences between ZT and CT, with CT porosity 47% higher than under ZT soils in the upper 10 cm when assessed by X-ray micro Computed Tomography (μCT) at a resolution of 64 μm. An increased number of macropores in CT compared to ZT in the surface but a higher proportion of micropores in ZT soils has been reported by Josa et al. (2013) and Mangalassery et al. (2014). Previously, most studies have focused on agricultural land under ZT management for ten years or fewer, with long-term studies typically viewed as those with 5 years beyond conversion. However, evidence suggests the duration of ZT management impacts both crop yield and soil properties. Díaz-Zorita et al. (2004) and Lal et al. (1994) identified significant changes in the structural properties in soils that had been under ZT for 20 and 28 years, respectively.

Many previous assessments of the impact of land management on soil porosity have previously been either qualitative or limited to the analysis of bulk samples with field structure disturbed or removed. X-ray μCT provides an alternative tool for measurement of structural properties in three-dimensions (3D) and in a non-destructive manner. Previously this technique has been applied to characterising soil hydraulic properties (e.g. Périard et al., 2016), quantifying the pore network structure (e.g. Baveye et al., 2002), quantifying seed-soil contact (e.g. Blunk et al., 2017) and to visualise undisturbed root architecture in soils (e.g. Tracy et al., 2010). We used X-ray μCT to assess and calculate the shape and size of pores as well as the connectivity and tortuosity of the pore network in soils that have been under ZT for 30 years in comparison to those ploughed annually. The aim was to reveal the differences in soil macroporosity, pore shape and pore size categories and pore connectivity for two important contrasting soil tillage systems and outline the implications for potential differences.

## 2 Material & methods

### 2.1 Experimental site

Soil samples were collected from a long-term experiment (~30 years) managed under both zero-till and conventional tillage systems. The experiment was carried out in Botucatu, São Paulo, Brazil (22^°^49′ S, 48^°^25′ W altitude: 786 m). The climate is classified as mesothermal with dry winters, and the dry season is well defined from May to September, with yearly average rainfall of 1450 mm, distributed mostly between October and April. The soil type is a Typic Rhodudalf (Soil Survey Staff, 2014) and the texture is clay uniformly in the soil profile (Soratto et al., 2014). The main soil chemical (Van Raij et al., 2001) and physical (EMBRAPA, 1997) properties are presented in Table 1. The cropping history included Wheat, Soybean, Black oat, Maize, Pearl millet, Dry beans, *Brachiaria* grass and Safflower (Table 2).

**Table 1:** Selected soil chemical and physical properties from the Botucatu site, prior to the establishment of the experiment.

pH (CaCl_2_)	Organic C	P	Al + H	Ca	Mg	K	CEC	BS	Sand	Silt	Clay
	(g kg^−1^)	(mg dm^−3^)	(mmolc dm^−3^)					(%)	(g kg^−1^)		
4.9	22	66	58	52	17	5.1	132	56	147	239	614

**Table 2:** Crop rotation history at the Botucatu experimental site.

Year	Crop rotation (fall-winter/spring-summer)
1985/86	Wheat/soybean
1986/87 to 1994/95	Wheat/soybean
1995/96 to 1998/99	Fallow/fallow
1999/2000	Black oat/maize
2000/01 to 2001/02	Fallow/fallow
2002/03 to 2003/04	Black oat/pearl millet-dry beans
2004/05 to 2005/06	Black oat/maize
2006/07	Fallow/soybean
2007/08	Yellow oat/dry beans
2008/09	Yellow oat/dry beans
2009/10 a 2011/12	Maize + *Brachiaria*/*Brachiaria*
2012/2013	*Brachiaria*/soybean
2013/2014	Wheat/soybean
2014/2015	Safflower/soybean
2015/2016	Safflower/maize
2016/2017	Black oat/maize
2017/2018	Maize/soybean

### 2.2 Soil sampling

Undisturbed soil samples were taken using a 12 cm length and 7.5 cm internal diameter cylinder, using a hydraulic system and a steel support to force the volumetric ring into the soil. Five cores were collected from an 8 × 40 m plot in each treatment (CT and ZT) using a randomized design. Since the five cores were collected in a single replicate of each treatment of the experiment, they comprise pseudo-replicates. The soil cores were sealed with paraffin wax at the top and bottom to prevent movement during transit and stored at 2 °C.

### 2.3 X-ray Computed Tomography (μCT)

Samples were packaged very carefully and transported to the Hounsfield Facility at the University of Nottingham for μCT scanning. Prior to this a pilot study involving soil samples being μCT scanned in Brazil and then μCT scanned again in the UK was undertaken to confirm that the addition of paraffin wax prevented any sample disturbance (see Supplementary Fig. 1). Samples were shipped to the UK as the μCT scanner available offered higher quality imaging. The soil cores were scanned using a G.E. V|Tomex|M Computed Tomography X-ray scanner. Each soil core was scanned for 45 min at 180 kV with an isotropic voxel size of 70 μm, with 2664 images per scan. The final image volume had 600 × 1400 × 600 voxels, corresponding to a volume of 42 × 98 × 42 mm.

The original grey-level X-ray μCT images were processed using ImageJ 1.42 software (http://rsbweb.nih.gov/ij/) after cropping to exclude the area outside the soil column. The segmentation process was based on the non-parametric Otsu method of automatic thresholding (Otsu, 1979). The images were also visually inspected to verify the quality of the segmentation procedure. This process resulted in a binary image, in which pores and soil solid material were respectively represented by white and black pixels. The distribution of greyscales in the image histogram was such that the organic material and pore space could be readily segmented separately.

For the 3D structure analysis, soil pores were classified according to their size and shape distribution. For the shape classification, geometrical parameters known as major, intermediate and minor axes of the ellipsoids that represent each pore were determined using 3D measuring techniques. These parameters were derived using the BoneJ plugin in the ImageJ 1.42 software. Binary images were utilized in these procedures. Isolated small pores <8 voxels were removed from the porous fraction of the images in order to avoid error associated with unresolved objects (Jefferies et al., 2014).

**Fig. 1:**
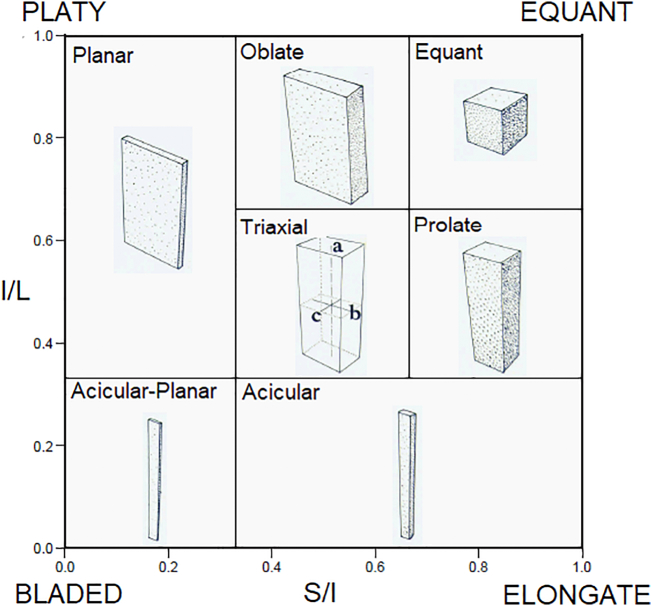
Soil pore classes based on the ratio of the principal ellipsoid axes. I: intermediate; L: large; S: short.

The soil pores which allowed the measurement of the three principal axes were classified according to the terminology suggested by Bullock et al. (1985). The relation between the ratio of the intermediate by the large (I/L) axis and the ratio of the short by the intermediate (S/I) axis allows the classification of pores based on shape (Fig. 1). The highest values of I/L and S/I ratios permit the classification of pores as spheres and the lowest ones as acircular-planar pores, for example. Therefore, the pores were classified as: Equant (Eq.), Prolate (Pr.), Oblate (Ob.), Triaxial (Tr.), Planar (Pl.), Acircular (Ac.) or Acircular-Planar (AP) (Pires et al., 2017; Ferreira et al., 2018). When one of the axes of a specific pore could not be determined by this approach due to their extreme complexity, they were considered non-classified pores (NC).

(Adapted from Bullock et al. (1985).)

The macroporosity (Ma) and the number of pores (NP) were calculated taking into consideration the image resolution. The 3D pore size distribution (sorted by pore volume) corresponded to the total number of disconnected volumes of pore space inside the total sample volume. For Ma and NP size distribution analysis, pores were classified in different volume intervals: 0.003–0.01; 0.01–0.1; 0.1–1; 1–10; 10–100; 100–1000 and >1000 mm^3^. These volume intervals were selected based on the importance of different pore sizes for water movement and retention.

The classification proposed by Brewer (1964) was also utilized to classify pore size. The classification was made based on the volume occupied by individual pores with specific equivalent cylindrical diameter (ECD). Pores with ECD smaller than 0.075 mm were considered to be mesopores, 0.075 to 1.0 mm were very fine macropores, 1.0 to 2.0 mm were fine macropores, 2.0 to 5.0 mm were medium macropores and >5.0 mm were coarse macropores. However, it is important to note that this classification was derived considering pores as cylindrical shaped entities (Hillel, 1998).

The network tortuosity (τ) of the pores and connectivity were calculated using the computer program, Osteoimage (Arcaro, 2013; Roque et al., 2009). Tortuosity was determined through the geodesic reconstruction algorithm implemented by Roque et al., 2012a, Roque et al., 2012b. Geometrically, tortuosity is defined as:

(1) $$\tau =\left(\frac{L_{G}}{L_{E}}\right),$$

where L_G_ and L_E_ represent the geodesic length between two connected points within the pore space and the Euclidian length between these two points. In this study, the average tortuosity was calculated considering three directions (x,y,z).

The Euler-Poincaré characteristic (EPC) was utilized to estimate the degree of connectivity, which represents one of the Minkowski functions and a topological measure used for describing the connectivity of spatial structures (Katuwal et al., 2015). This parameter for a 3D structure is related to the number of isolated parts minus the connectivity of an object (Thurston, 1997). EPC values with respect to the contiguous image sections, known as disectors (Sterio, 1984), were obtained considering 599 disectors. Based on these values the EPC per sample volume was evaluated (Roque et al., 2012b). Therefore, the EPC number is an indicator of how connected a pore is: the smaller (more negative) it is, the higher the pore connectivity (Chappard et al., 1999; Roque et al., 2009).

### 2.4 Statistical analysis

The statistical analysis was performed using the R software (R version 3.3.3; R Core Team, 2017). Since the soil cores represented pseudo-replicates, a non-parametric test, Kruskal–Wallis, was used to test the effects of the two tillage management systems on macroporosity (Ma), number of pores (NP), Euler-Poincaré characteristic (EPC) and network tortuosity in each axis (τ), considering a p < 0.05 significance level.

## 3 Results

**Fig. 2:**
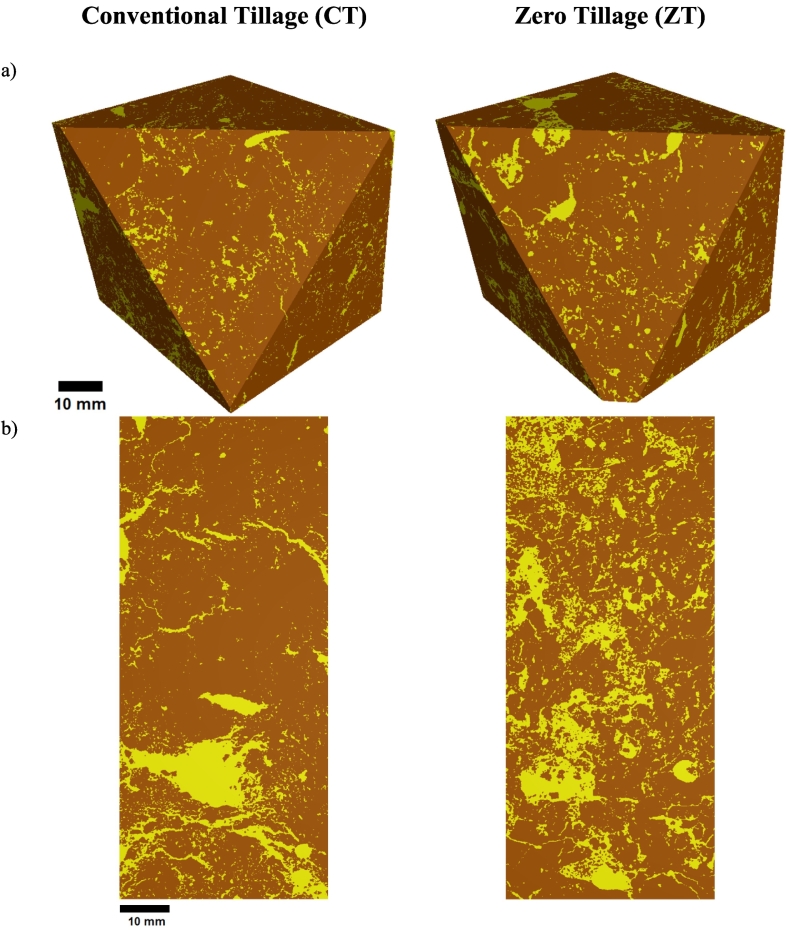
Microtomographic images for the soil under conventional tillage (left column) and zero tillage (right column) for the whole soil volume orthogonal (a) and vertical (b) cross-sections.

**Fig. 3:**
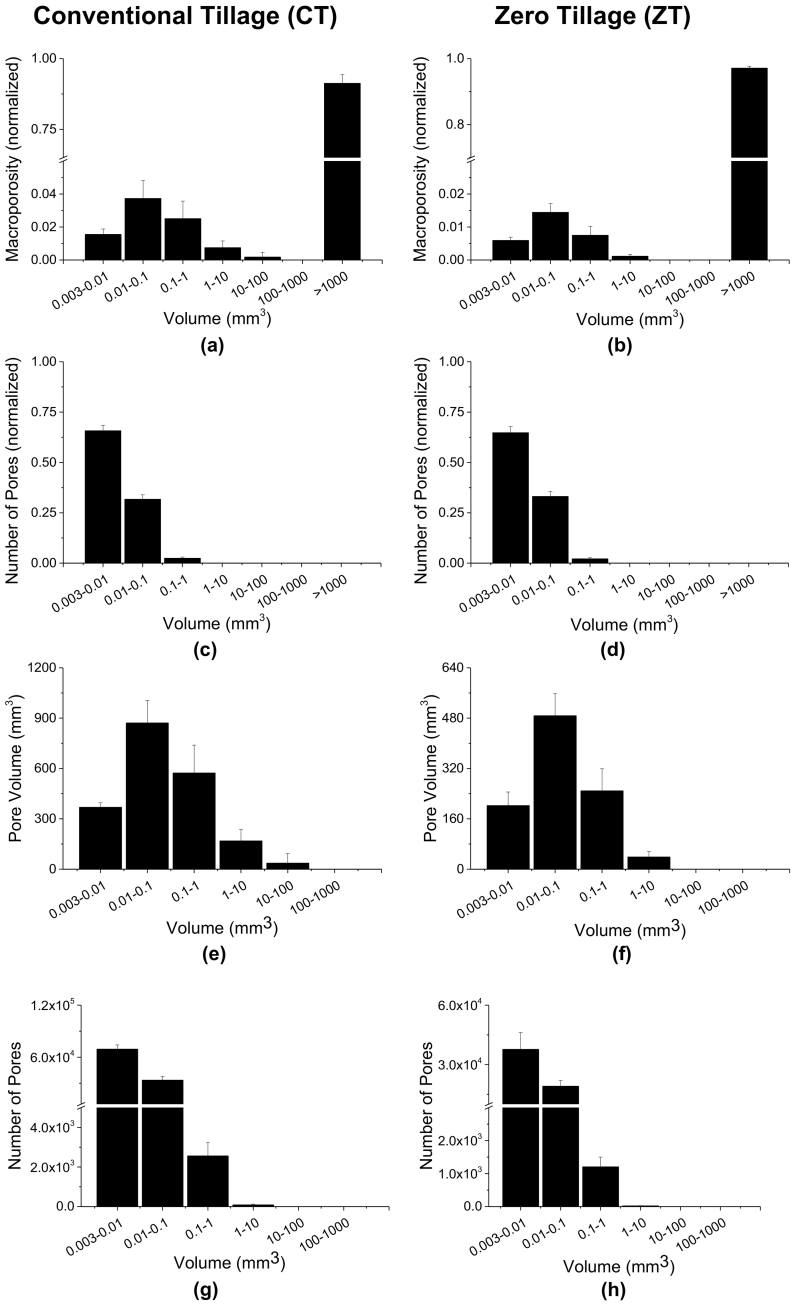
Pore classification according to size for a soil under CT and ZT. Macroporosity and number of pores for each size class were normalized based on the total macroporosity and the total number of pores.

Fig. 2 presents 3D images of the soil porous system of one representative sample under CT and ZT. For ZT, well-defined branches of connected pores can be clearly observed while for CT, the macroporosity is represented by a more random structured, agglomeration of pores. The contribution of different pore volumes to Ma and NP, for volume of pores >8 voxels, is presented in Fig. 3. The exclusion of the pore volumes <8 voxels from the segmented images only marginally affected the soil macroporosity (difference of 0.2% for CT and 0.1% for ZT). The average Ma obtained for the soil under CT was 14.3% (±3.6%) and the average NP was 105,539 (±8780). For soils under ZT, the average Ma was 19.7% (±1.9%) and the average NP was 58,022 (±10,968).

The results presented in Fig. 3 indicate that there was a significant contribution (≈91%) of one single large pore (>1000 mm^3^) to Ma in CT, which is associated with the connectivity of the pore system (Fig. 3a). This was even more pronounced in ZT, with one single large pore (>1000 mm^3^) accounting for 97% of Ma (Fig. 3b), indicating a highly connected pore network. The contribution of pores between 0.003 and 100 mm^3^ to Ma in both treatments is small (<10%) as observed in Fig. 3a and b, but these pore sizes presented the greatest contribution to NP (≈99%) (Fig. 3c, d). In both CT and ZT, pore sizes between 0.003 and 0.01 mm^3^ contributed to around 65% of NP (Fig. 3c, d).

When the largest pore was excluded from the analysis (Fig. 3e–h), pores with sizes between 0.01 and 1 mm^3^ had the greatest contribution to Ma. The average sum of pore volumes was 871 (0.01–0.1 mm^3^) and 573 mm^3^ (0.1–1 mm^3^) in CT (Fig. 3e). ZT presented lower values with 488 (0.01–0.1 mm^3^) and 249 mm^3^ (0.1–1 mm^3^) (Fig. 3f). The highest concentration of pores was found for the size interval of 0.003 to 0.01 mm^3^ with 69,297 pores in CT and 37,722 pores in ZT (Fig. 3g, h).

**Fig. 4:**
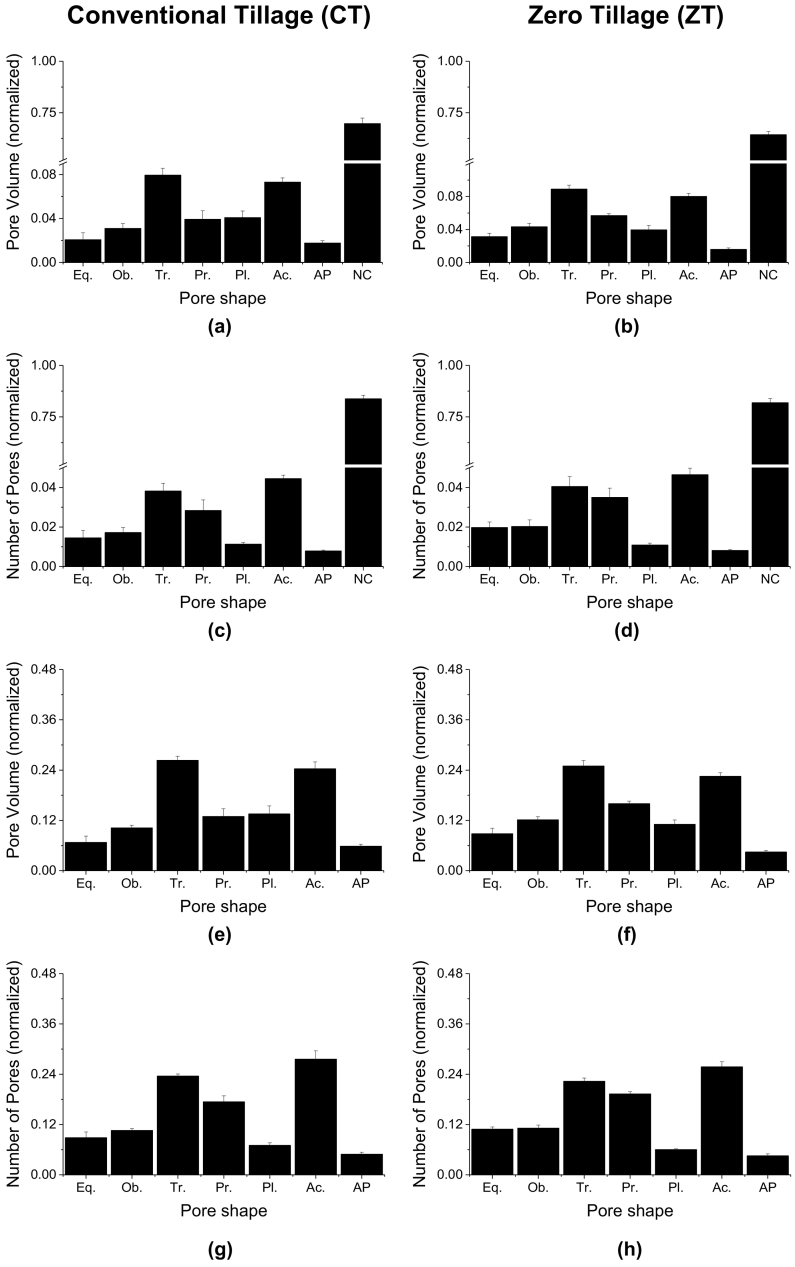
Pore classification according to shape for a soil under CT and ZT. Eq: equant; Ob: oblate; Tr: triaxial; Pr: prolate; Pl: planar; Ac: acircular; AP: acircular-planar. Pore volume and number of pores for each shape were normalized based on the total pore volume and the total number of pores.

The contribution of the shape of pores to P and NP is presented in Fig. 4. For soils under CT, the greatest pore volume belonged to the non-classified (i.e. of extreme complexity) pore class (1402 mm^3^) followed by the triaxial (161 mm^3^) and acircular (149 mm^3^) shapes (Fig. 4a). Similarly, the greatest pore volume in ZT belonged to non-classified pores (629 mm^3^) followed by the triaxial (87 mm^3^) and acircular (78 mm^3^) shaped pores (Fig. 4b). The contribution of NC pores in the total volume was similar in CT (70%) and ZT (64%), as presented in Fig. 4a and b. In terms of the NP (Fig. 4c, d), the percentage of NC pores was also similar in CT and ZT, with 82% and 84%, respectively.

When NC pores were excluded from the analysis in the CT treatment (Fig. 4e, g), triaxial and acircular shapes represented around 29 and 26% of the volume of pores, respectively. In terms of NP, the latter contributed with 32% and the former 28%, respectively. For the ZT pore types, triaxial and acircular shapes represented around 27 and 24% of the volume of pores respectively when NC pores were not considered (Fig. 4f,h). In terms of NP, the latter had a contribution of around 29% and the former 25%, respectively. These results indicate that rod- (acircular) and cuboid-shaped (prolate and triaxial) pores are the most common for CT when the very complex pores are excluded. However, it is important to mention that the contribution of classified pores to Ma and NP was around 36% and 18% (ZT) and 30% and 16% (CT) between tillage systems.

**Fig. 5:**
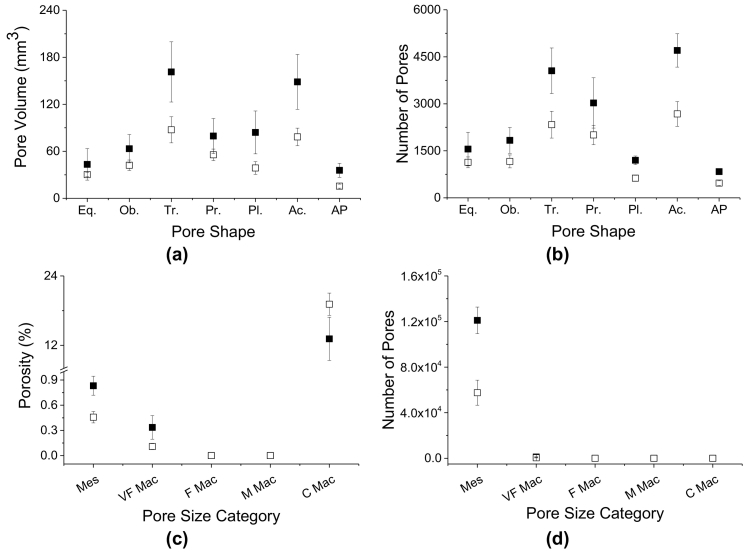
Comparison between conventional tillage (CT, ■) and zero-tillage (ZT, □) of pore volume by shape (a), number of pores by shape (b), porosity (%) by pore size category (c) and number of pores by pore size category (d). Mes: mesopore; VF Mac: very fine macropores; F Mac: fine macropores; M Mac: medium macropores; C Mac: coarse macropores.

Soils under CT had the largest pore volumes when different pore shapes were considered (Fig. 5a), and a larger number of pores (Fig. 5b) than ZT. The single largest pore, included in the coarse macropore category, in ZT was responsible for the larger average macroporosity compared to CT (Fig. 5c). The larger number of mesopores in CT as compared to ZT is also apparent in Fig. 5d.

The pore connectivity result indicated that soils under ZT (EPC = 297) were more connected than soils under CT (EPC = 931) (Table 3). A smaller value for EPC relates to a more connected pore system. The soil under CT had the largest average values of tortuosity in all directions (τ_x_ = 1.48, τ_y_ = 1.46, τ_z_ = 1.48) compared to tortuosity in ZT (τ_x_ = 1.35, τ_y_ = 1.33, τ_z_ = 1.35). The average tortuosity considering the three directions was 1.47 and 1.34 for CT and ZT, respectively.

**Table 3:** Macroporosity (Ma) by image, total number of pores (NP), Euler-Poincare characteristic (EPC) and tortuosity (τ).

Parameter	Mean	SD	Max	Min
Conventional tillage (CT)				
Ma (%)	14.3^b^	3.6	20.2	11.4
NP	105,539^a^	8780	117,267	94,617
EPC	931^a^	124	1040	727
τ_+x_	1.50^a^	0.09	1.58	1.40
τ_−x_	1.47	0.10	1.57	1.35
τ_+y_	1.46^a^	0.13	1.61	1.34
τ_−y_	1.46^a^	0.13	1.68	1.35
τ_+z_	1.45	0.10	1.52	1.29
τ_−z_	1.51^a^	0.12	1.71	1.44

Zero-tillage (ZT)				
Ma (%)	19.7^a^	1.9	22.4	17.6
NP	58,022^b^	10,968	72,145	45,514
EPC	297^b^	198	630	143
τ_+x_	1.34^b^	0.02	1.37	1.31
τ_−x_	1.35	0.02	1.37	1.32
τ_+y_	1.34^b^	0.04	1.41	1.30
τ_−y_	1.32^b^	0.06	1.42	1.29
τ_+z_	1.34	0.04	1.40	1.31
τ_−z_	1.35^b^	0.02	1.38	1.33

Mean values followed by different letters differed significantly at p < 0.05 between tillage systems.

## 4 Discussion

These findings show that the porous system in both tillage systems for this soil type is characterised by highly connected pores. The effect of the conventional management contributed to an increase in the number of separate pore entities, with CT presenting almost twice the number of pores as ZT. This is mainly associated with the changes in the soil structure caused by the ploughing operations, which contribute to the disaggregation of the structure at the top soil (Bauer et al., 2015). These operations contribute mainly to increasing the amount of small pores that are important for water storage in particular explaining why ploughing is often viewed necessary by farmers. Tavares Filho and Tessier (2009), assessing differences in CT and ZT in a Brazilian Oxisol, observed a disaggregated microaggregate structure between 0 and 20 cm for CT compared to the presence of fissures and biopores (which typically develop over longer time scales) for ZT. Similarly, Lipiec et al. (2006) reported well-defined aggregates separated by large irregular cavities in CT and poorly defined aggregates with more circular pores under ZT.

An increase in soil porosity, especially in macroporosity, due to tillage has been frequently reported (e.g. Mangalassery et al., 2014; Pöhlitz et al., 2018). De Moraes et al. (2016) observed higher macroporosity in CT treatment in the surface layer (0–10 cm depth) compared to long-term (11 and 24 years) treatments under ZT in a clay Oxisol in Southern Brazil. However, at depths below 10 cm, CT reduced macroporosity to levels likely to be critical for crop growth, most likely due to the formation of a harrow pan resulting from a continuous use of a heavy disk harrow in the CT treatment. Lipiec et al. (2006) found greater areal and stained soil porosity in CT over long term ZT in a silt loam Eutric Fluvisol in Poland, using resin-impregnated soil blocks. The opposite was observed by Pires et al. (2017) for a Brazilian Rhodic Ferralsol of clay texture under long term ZT, using X-ray CT with an image acquisition resolution of 20 μm. Using a combination of mercury intrusion porosimetry and X-ray CT, Piccoli et al. (2017) concluded that a conservation agricultural system comprised of no-tillage, cover crops and residue retention in silty soils in the Veneto Region in Italy did not significantly change macroporosity (>26 μm), but increased ultramicroporosity (0.1–5 μm), compared to conventional intensive tillage after five years of implementation. The authors attributed the slow reaction to conservation agriculture in macroporosity to poor aggregate stability and low soil organic carbon content in the studied soils. Comparing soil under ZT and CT for six years in an Oxisol in Brazil using the tension table method, Stone and Silveira (2001) found higher total and macroporosity in CT, and higher microporosity in ZT. This supports the hypothesis that porosity at the microscale develops over longer timescales than at the macroscale where the impacts of tillage are observed more rapidly (Bacq-Labreuil et al., 2018). An important consideration in this study is the duration of time that the soils had been managed under ZT. Very few previous studies have examined the soil structure following as long as 30 years of ZT and it is most likely this reason that we report a significant increase in macroporosity and connectivity. We hypothesise that the undisturbed faunal and floral activity in the soil over a significant time scale is responsible for this result. Following on from this, it seems likely these effects might be more apparent at assessment scales below the 70 μm considered here.

The comparison of the average Ma and NP between tillage systems demonstrates that the ploughing operation in the case of CT presents a high variability among samples in terms of Ma (CVs of around 25% - CT and 10% - ZT). On the other hand, this management system has the lowest variability in the amount of pores (CVs of around 8% - CT and 19% - ZT). This is mainly related to the homogeneity in soil structure that results from the preparation after ploughing operations as is required for seedbed preparation (Garbout et al., 2013). Ploughing operations tend to destroy the aggregates, especially those larger sized, often referred to as clods, homogenizing the soil (Torre et al., 2018). Although ploughing is used as a way to reduce compaction, this effect is related to the rupture of aggregates, which increases soil fragility due the re-accommodation of soil particles. The effect of this mechanical de-compaction of the soil is ephemeral, because the different forces that the soil undergoes result in a fast re-accommodation of the particles and increases soil penetration resistance (Calonego et al., 2017).

The soil under CT had larger pore volumes when the different pores were classified in terms of shape were considered, and a larger NP than ZT, which can be associated to aggregate breakdown induced by ploughing. Borges et al. (2018) also found an increase in NP for a Brazilian clay Oxisol under CT in relation to ZT which they attribute to aggregate breakdown creating a higher number of pores under CT at the topsoil (0–10 cm). Soils with a large number of disconnected pores are normally characterised by large tortuosity values, which was confirmed by the values for CT (Borges et al., 2018), and likely to have reduced hydraulic conductivity and gaseous diffusion in comparison to soils with higher pore connectivity. Ferreira et al. (2018) observed for a Brazilian Dystrudept silty-clay soil that residual pores are characterised by high tortuosity values when compared to connected porous systems, as observed in our work. We found the soil under long-term ZT had lower EPC values, which indicates better pore connectivity. Borges et al. (2018) demonstrated that the top soil layer (0–10 cm) of a Brazilian clay Oxisol under long-term ZT was characterised by a more developed pore connectivity than CT. We hypothesise this could be due to the impact of the root system being allowed to remain in-situ and decay over several years and the undisturbed activity of the soil fauna, in particular earthworms. Soils under long-term ZT usually have higher bulk density values and lower porosity (mainly macroporosity) as compared with CT, as found by Ismail (2011), Zaraee and Afzalinia (2016) and Table 1 in Supplementary Material. However, under long-term ZT there is an absence of studies reporting physical restriction to root growth (Busari et al., 2015). The absence of restriction to root growth is likely due to the presence of biopores produced by macro and mesofauna organisms or by the decomposition of the root system of previous crops and cover crops, which is difficult to quantify. A study by Mangalassery et al. (2015) compared sixty published experiments with paired ZT and CT treatments and found that approximately half reported yield advantage under ZT and the other half reported yield advantage under CT. It is likely that significant differences exist depending on soil type, climate and cropping system.

Higher pore connectivity in ZT could enhance root penetration through a reduction in foraging and enhanced water and nutrient availability which might explain why high yields have been reported in some long-term ZT systems (Pittelkow et al., 2015; Busari et al., 2015). This effect would be likely to be more pronounced in drier years (Calonego and Rosolem, 2010), even in soils with high bulk density and resistance to penetration. Therefore, it is evident that conventional evaluations of soil physical properties, measuring standard, quantitative characteristics are not idea in isolation to quantify the true physical state of the soil, and investigations that consider soil morphology are also needed to provide a better understanding of the effects of the conservation tillage systems in root growth and yield potential of crops. We attribute the differences in soil porosity reported in this study to the influence of the long-term implementation of the CT and ZT treatments (close to 30 years). Soil structural changes and related soil organic matter dynamics, in addition to the impact of the soil faunal community under ZT are more pronounced over long-term ZT implementation. In a review focusing on the effect of conservation tillage on soil porosity and organic matter, Kay and VandenBygaart (2002) concluded that the most consistent results concerning ZT are obtained when measurements were made at or above 15 years after conversion, though currently few comparison studies over these timescales are available.

The pore connectivity analysis indicated that the soil under ZT (EPC = 297) is more connected that the soil under CT (EPC = 931). Soil under CT presented larger values of tortuosity (τx = 1.48, τy = 1.46, τz = 1.48) than ZT (τx = 1.35, τy = 1.33, τz = 1.35). These results indicate that long-term adoption of ZT leads to higher pore connectivity and greater macroporosity, which has implications in nutrient cycling, root growth, soil gas fluxes and water dynamics. We suggest that considering the wide range of morphological properties used to characterise soil structure, which is increasingly accessible as the use of image analysis software becomes more prevalent, that the soil pore connectivity is one, if not the most important measurements in this regard. Previous studies have suggested porosity is significantly reduced in the short term following conservation tillage practices (e.g. Mangalassery et al., 2014), so it is important that we here highlight the positive effects in soil structure observed in this study are most likely related to the long-term nature of the ZT implementation. This is especially important in the context that while >50% of crop land in Brazil is managed as ZT or conservation agriculture (de Freitas and Landers (2014), it is much less prominent in other parts of the world e.g. Europe <5% (ECAF, 2017).

## 5 Conclusions

X-ray Computed Tomography was useful in assessing qualitative and quantitative differences between long-term conventional tillage and zero-tillage systems in soil pore volume, structure, connectivity, tortuosity, size and shape distribution.

Soils under long-term zero-tillage present better pore connectivity and higher total porosity than conventional tillage. Long-term conventional tillage tends to increase the total number of pores compared to the zero-tillage system, which is likely related to changes in the soil structure caused by the ploughing operations.

## Appendix A Supplementary data

Supplementary material Image 1
