# Supplementary material for: Assessing the long-term effects of zero-tillage on the macroporosity of Brazilian soils using X-ray Computed Tomography
Source: Geoderma. 2019 Mar 1;337:1126–35. doi: 10.1016/j.geoderma.2018.11.031 (PMC6358041; doi:10.1016/j.geoderma.2018.11.031)
Supplement: Supplementary file 1 — Supplementary material [file mmc1.docx]

Supplementary Material


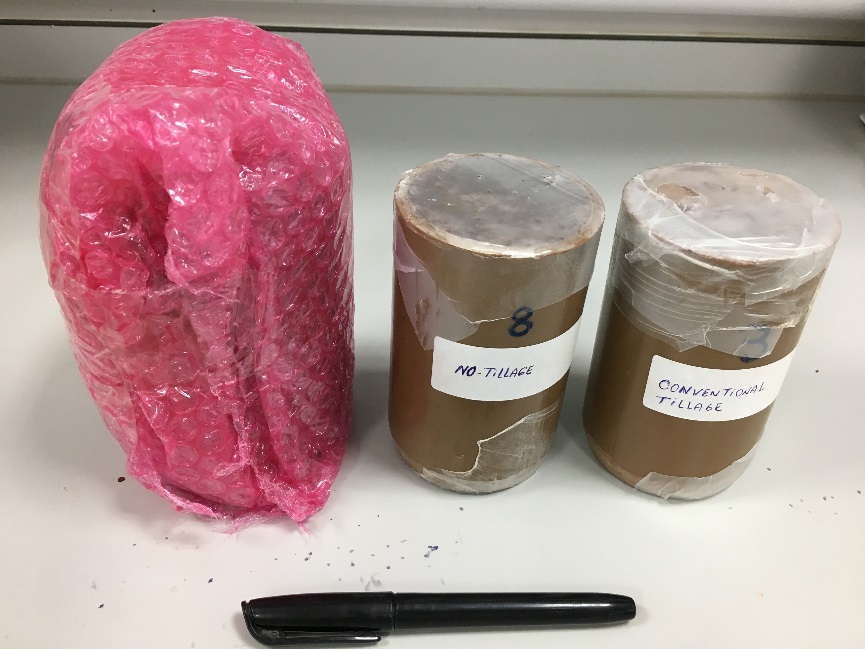


Figure 1. Soil core samples with paraffin wax and wrapping material for shipping for X-ray Computed Tomography scanning.

Figure 2. Soil water matric potential in the Conventional Tillage (CT) and Zero-Tillage (ZT) treatments at 0-5 cm depth.

Figure 3. Soil water cumulative infiltration (cm) in the Conventional Tillage (CT) and Zero-Tillage (ZT) treatments.

Figure 4. Soil water infiltration velocity (cm h^-1^) in the Conventional Tillage (CT) and Zero-Tillage (ZT) treatments.

Table 1. Soil Bulk Density (BD) in Conventional Tillage (CT) and Zero-Tillage (ZT) treatments by depth (cm). CV: Coefficient of Variation.

| Tillage  System | BD |
| --- | --- |
|  | (Mg m^-3^) |
|  | **0 - 5 cm** |
| CT | 1.03 |
| ZT | 1.16 ns |
| *CV* | *15.84* |
|  | **5 - 10 cm** |
| CT | 1.13 |
| ZT | 1.26 * |
| *CV* | *14.08* |
|  | **10 - 20 cm** |
| CT | 1.25 |
| ZT | 1.34 * |
| *CV* | *12.18* |

* different by the F test at 5% probability
